# Application of different types of CRISPR/Cas-based systems in bacteria

**DOI:** 10.1186/s12934-020-01431-z

**Published:** 2020-09-03

**Authors:** Zhenquan Liu, Huina Dong, Yali Cui, Lina Cong, Dawei Zhang

**Affiliations:** 1 School of Biological Engineering, Dalian Polytechnic University, Dalian, 116034 People’s Republic of China; 2 Tianjin Institute of Industrial Biotechnology, Chinese Academy of Sciences, Tianjin, 300308 China; 3 Key Laboratory of Systems Microbial Biotechnology, Chinese Academy of Sciences, Tianjin, 300308 People’s Republic of China; 4 University of Chinese Academy of Sciences, Beijing, 100049 China

**Keywords:** CRISPR/Cas system, Genome editing, Bacteria

## Abstract

As important genome editing tools, CRISPR/Cas systems, especially those based on type II Cas9 and type V Cas12a, are widely used in genetic and metabolic engineering of bacteria. However, the intrinsic toxicity of Cas9 and Cas12a-mediated CRISPR/Cas tools can lead to cell death in some strains, which led to the development of endogenous type I and III CRISPR/Cas systems. However, these systems are hindered by complicated development and limited applications. Thus, further development and optimization of CRISPR/Cas systems is needed. Here, we briefly summarize the mechanisms of different types of CRISPR/Cas systems as genetic manipulation tools and compare their features to provide a reference for selecting different CRISPR/Cas tools. Then, we show the use of CRISPR/Cas technology for bacterial strain evolution and metabolic engineering, including genome editing, gene expression regulation and the base editor tool. Finally, we offer a view of future directions for bacterial CRISPR/Cas technology.

## Introduction

Bacteria have rapid reproduction rates, are metabolically diverse, and can produce complex molecules that cannot be produced through conventional chemical syntheses, such as enzymes and a myriad secondary metabolites [1]. With the development of metabolic engineering, many high-yield strains for industrial production have been established [2–4]. Bacterial cell factories have broad development prospects in industrial production. The development of genetic engineering tools is very important for the application of bacteria in modern industrial production. In recent years, clustered regularly interspaced short palindromic repeats (CRISPR)/CRISPR-associated protein (Cas) systems were widely used for genetic engineering of bacteria, which has greatly promoted their application.

According to the structure and function of Cas protein, the CRISPR/Cas systems can be categorized into two classes (class I, class II), which are further subdivided into six types (type I–VI) [5]. Class I includes type I, III, and IV, and class II includes type II, V, and VI [6]. Type I, II, and V systems recognize and cleave DNA, type VI can edit RNA, and type III edits both DNA and RNA. How the effect of type IV system on DNA or RNA is still unknown [7]. Studies have shown that all CRISPR/Cas systems may be derived from the same ancestor, whereby class I was encoded by a single-function *cas* gene, and lost a portion of the additional *cas* gene during evolution to form class II [6]. Since the structures of type II and V systems are relatively simple, they have been widely used in bacteria. The development of endogenous type I and III systems has expanded the use of CRISPR/Cas technology in bacteria.

In this review, we summarize the mechanisms of CRISPR/Cas systems and analyze their similarities and differences. Then, the existing applications are classified and summarized according to genome editing, CRISPR interference (CRISPRi), and the base editor tool. In addition, by drawing attention on the newly developed CRISPR/Cas tools in eukaryotes, we offer ideas on how to optimize and develop new genome editing tools in bacteria in future studies.

## Mechanisms of different CRISPR/Cas systems

**Fig. 1:**
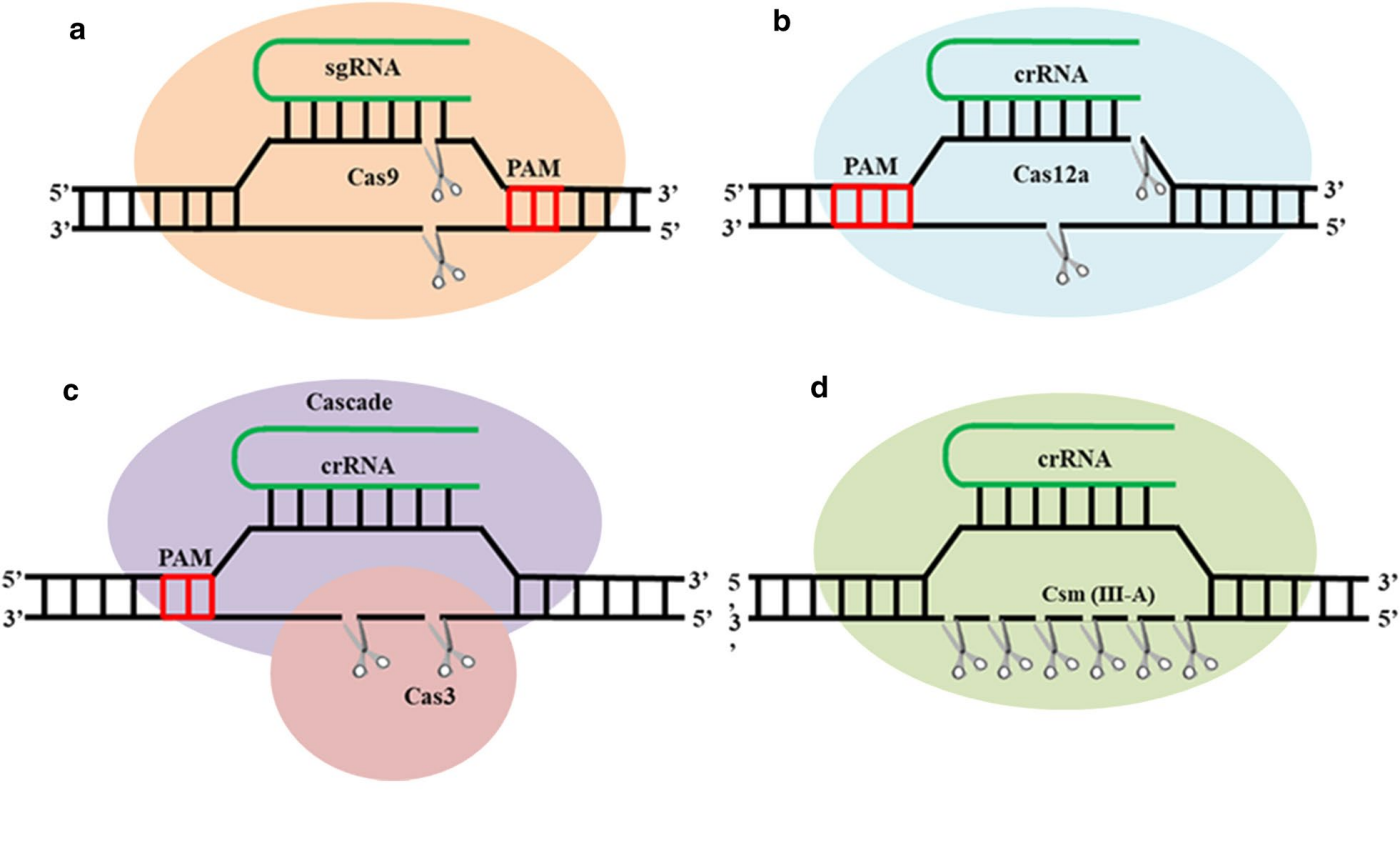
Schematic of the mechanisms of different types of CRISPR systems. **a** The working principle of type II Cas9. In the presence of the PAM sequence (NGG), the targeting effect of sgRNA is used to guide Cas9 protein to cleave both the complementary and non-complementary strands, forming a blunt-ended nick. **b** The working principle of type V Cas12a protein. In the presence of the PAM sequence (NTTT), the targeting effect of crRNA is used to guide Cas12a protein to cleave both the complementary and non-complementary strands, forming a sticky-ended nick. **c** The working principle of type I Cas systems. In the presence of the PAM sequence, the targeting effect of crRNA is used to guide the Cas3 protein to cleave the non-complementary strand to form a large gap. **d** The working principle of type III Cas systems. In the absence of a PAM sequence, the targeting effect of sgRNA is used to guide Csm protein to cleave the non-complementary strand to form short nucleic acid fragments. The green transverse U represents sgRNA or crRNA, the nucleotide sequences marked in red represent the PAM sequence, and scissors represent the cleavage site of nucleases

As Jinek et al. [8] first demonstrated, the CRISPR/Cas9 system specifically cleaves double-stranded DNA (dsDNA) in vitro and leads to double-strand breaks (DSBs), indicating that it can be used for genome editing. CRISPR/Cas9 has the advantages of high efficiency, simple design, and simple operation, so it plays an important role in genome editing. As research progressed, many new CRISPR/Cas systems have been discovered and used for genome editing, including the type V Cas12a [9] system, as well as the endogenous type I [10] and III [11] CRISPR/Cas systems. All these systems have their own characteristics, such as different protospacer adjacent motif (PAM) regions, different Cas protein sizes, and different cleavage sites, which are summarized in Fig. 1.

The type II CRISPR/Cas9 genome editing system comprises Cas9 protein, CRISPR RNA (crRNA) and trans-activating crRNA (tracrRNA). The currently used Cas9 protein, containing 1368 amino acids, encompasses a REC (recognition lobe) and a NUC (nuclease lobe). The NUC domain contains a highly conserved RuvC nuclease domain and an HNH nuclease domain. The former cleaves the same single strand (non-complementary strand) as the protospacer sequence, while the latter cleaves a single strand complementary to the crRNA sequence. Simultaneously, both of them act at a specific position in the target sequence to produce a blunt end [12]. The PAM region of Cas9 is at the 3′ end of the target sequence, and its sequence is 5′-NGG-3′.

TracrRNA is a hairpin RNA transcribed from a repeat region. TracrRNA, precursor crRNA (pre-crRNA) and Cas9 protein form a complex in which tracrRNA is responsible for activating RNase III to promote the maturation of the pre-crRNA [13]. Mature crRNA combines with tracrRNA and Cas9 to activate cleavage. A single-stranded guide RNA (sgRNA), a fusion of crRNA and tracrRNA, can effectively recognize specific sequences and direct the action of Cas9 protein [8], which greatly simplifies the process of genome editing.

The type V CRISPR/Cas12a genome editing system comprises crRNA and Cas12a protein. The Cas12a protein contains a RuvC endonuclease domain, which sequentially cleaves the non-targeting strand and the targeting strand to form DSBs [14]. Compared to the CRISPR/Cas9 system, this system has a number of remarkable differences, including the signature protein, PAM sequence and cleavage product (Table 1).

**Table 1:** Differences between type I, II, III, and V CRISPR/Cas systems

Classification	Type I	Type II	Type III	Type V
Signature protein	Cas3 (or Cas3′)	Cas9 (1368 amino acids)	Csm (III-A) or Cmr (III-B)	Cas12a (1200–1300 amino acids)
Effector	Cascade	crRNA and tracrRNA (sgRNA)	Cascade	crRNA
PAM sequence	3-nt	G-rich sequence, 5′-NGG-3′	Without PAM	5′-YTN-3′(FnCas12a), 5′-TTTN-3′(AsCas12a, LbCas12a)
Cleavage product	SSBs	DSB (flat end)	SSBs at every 6-nt	DSB (Sticky end with 5 nucleotides protruding)

Type I systems have the most *cas* genes, which are encoded by one or more operons. They contain six proteins, including the Cas3 protein which has helicase and nuclease activities, and is the main enzyme in the interference phase. Multiple Cas proteins are combined with mature crRNA to form a CRISPR-associated complex for antiviral defense (Cascade), which binds to invading foreign DNA and promotes the pairing of crRNA and the complementary strand of exogenous DNA to form an R loop, which is recognized by Cas3 to cleave both the complementary and non-complementary strands.

Type III systems contain the Cas10 protein with RNase activity and Cascade, and the function of Cascade resembles type I systems. Cas10 protein plays an important role in the maturation of crRNA and cleavage of invading foreign DNA. Type III systems are categorized into four subtypes named A–D. The interference target of type III-A is mRNA, while the interference target of type III-B is the same as that of type I and II CRISPR/Cas systems, which is DNA. However, the interference targets of types III-C and D are unclear.

Furthermore, the ribonucleoprotein complexes of type II and V systems are relatively simple compared with those of types I and III. Type II systems only require crRNA, tracrRNA, and Cas9 protein. The even simpler type V systems only require crRNA and Cas12a protein.

## Applications of type II CRISPR/Cas systems in bacteria

Type II CRISPR/Cas systems are characterized by a signature component, like the Cas9 protein, which includes three subtypes, II-A, II-B, and II-C. The CRISPR/Cas9 system, designed as a genome-editing tool, encompasses Cas9 protein which can cleave dsDNA at the target sequence with high specificity, and the sgRNA which recruits Cas9 to the target site [15]. Cas9 induces DSBs by cleaving the DNA single strand paired with the 20-bp sgRNA via the HNH nuclease domain and the other DNA strand via the RuvC domain [8]. The DSBs produced by Cas9 can be repaired by the non-homologous end joining (NHEJ) pathway or the homology-directed repair (HDR) system. A combination of Cas9 protein with DNA repair pathways can be used for gene deletion or insertion in the bacterial genome [16].

**Fig. 2:**
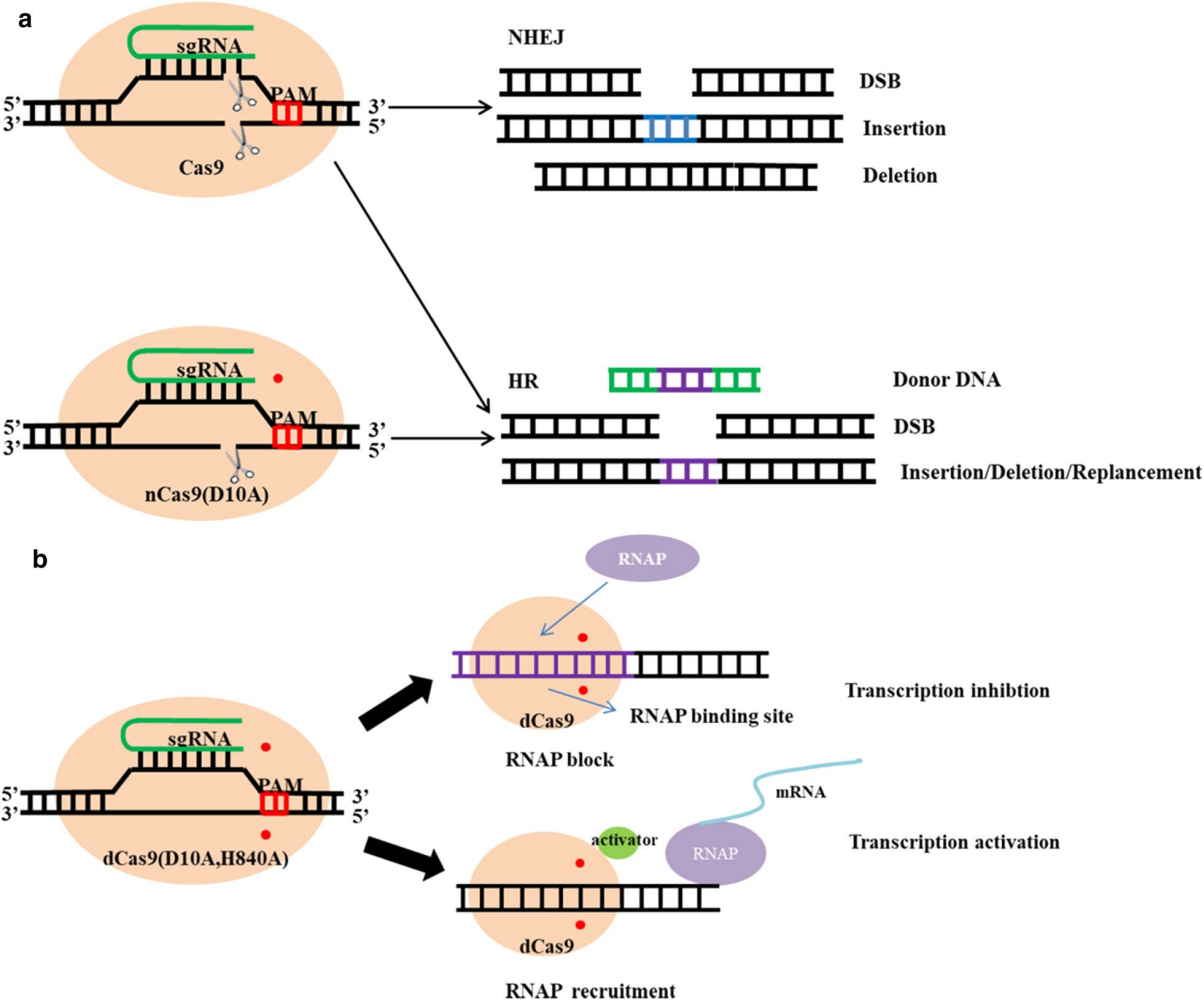
Schematic of the gene tools based on Cas9, nCas9, and dCas9, respectively. **a** The HR system is used to accurately repair the DSBs introduced by Cas9 protein or the single-strand breaks introduced by nCas9 protein when donor DNA fragments are provided. The NHEJ system is used to inaccurately repair the DSBs introduced by Cas9 protein when Donor DNA fragments are not provided. **b** The combination of dCas9 and the target site inhibits the combination of RNAP and the DNA strand, which weakens the DNA transcription process and reduces the expression of the targeted gene. The expression of a fusion protein comprising dCas9 and a transactivator domain can recruit RNAP and enhance the process of DNA transcription, thus increasing targeted gene expression. The red dots in A/B represent gene mutations in the corresponding cleavage domains

The Cas9 nickase (nCas9), which differs from Cas9 protein in a single point mutation (D10A or H840A), can also cleave single-stranded target sites recognized by sgRNA. When repair systems fail to repair the DSBs introduced by Cas9, which causes bacterial cell death, it can be used for genome editing instead of Cas9 [17]. However,nCas9 still retains a cleavage domain, which can perform single-stranded cleavage of the genome. In some strains with the less efficient native DNA repair mechanism and the invalid external repair mechanism, nCas9 can easily cause cell death, which may limit its use in bacteria [18] (Fig. 2).

In addition, researchers produced H840A and D10A double mutants of the HNH and RuvC domains of Cas9 protein, respectively, to inactivate the endonuclease and form dead Cas9 (dCas9) [8]. The resulting dCas9 protein can be fused to transcriptional activators to produce the CRISPR activation (CRISPRa) system, which recruits RNA polymerase (RNAP) to induce transcription [19]. It can also be used to bind to specific genomic loci to effectively inhibit the transcription of downstream genes [20]. This system, called CRISPRi, can selectively regulate the expression of a target gene guided by sgRNA. In bacteria, CRISPRi is a more preferred transcriptional regulation tool for gene knockdown than RNA interference (RNAi). Bacterial CRISPRi has already been developed into a mature technology with many applications [21–24], whereas bacterial CRISPRa has been successfully applied in only a few reports [19, 25]. Additionally, dCas9 can be fused with a deaminase to produce a base editor, which relies on targeting by dCas9 and deamination by deaminase to induce base conversions at the target site [18, 26].

### CRISPR/Cas9-mediated genome editing

#### CRISPR/Cas9 and HDR-mediated genome editing

In traditional methods, the λ-Red recombination system derived from λ phage was the most widely used, and it could efficiently integrate foreign DNA carrying homologous sequences into the bacterial genome, but generally required about a week per insertion [27]. In order to shorten the editing time, the CRISPR/Cas9 system and λ-Red recombination system were co-expressed, enabling the introduction of gene knockouts, insertions or substitutions on the *E. coli* chromosome without a selectable marker gene, thereby omitting the work needed for the deletion of the marker and greatly shortening the editing time [28–30].

In order to further shorten the experimental procedure, the genes encoding Cas9, sgRNA, λ-Red recombinase and templates were integrated into the same plasmid, which shortened the genome editing cycle to 3 days [31]. Similarly, a time-saving CRISPR/Cas9 system (pCasSA) was developed for knockout, knock-in and single-base mutations in *Staphylococcus aureus* [32].

In order to further improve the efficiency of genome editing, CRISPR/Cas9 and λ-Red recombinase-based MAGE technology (CRMAGE) was established, which could edit three genes simultaneously in *E. coli* with a recombination efficiency between 96.5 and 99.7%. By contrast, the efficiency of traditional recombination systems was only between 0.68 and 5.4% [33]. The researchers used the plasmid pKCcas9dO encoding a codon-optimized cas9, two homology-directed repair templates, and a target-specific guide RNA to successfully achieve gene editing in *Streptomyces coelicolor* M145 with efficiencies of 60–100% [34]. The editing efficiency of the CRISPR/Cas9 tool specifically optimized for *E. coli* was nearly 100%, and it could be used to induce three mutations simultaneously [35].

In order to develop a Cas9 protein adapted to a broader temperature range, ThermoCas9 was developed in *Geobacillus thermodenitrificans* T12. ThermoCas9 was active between 20 and 70 °C in vitro and was successfully used in vivo for gene knockouts and silencing in *Bacillus smithii* at 55 °C, as well as in *Pseudomonas putida* at 37 °C [36].

Furthermore, in order to solve the problem of editing large DNA fragments, researchers developed a three-plasmid system that could delete up to 19.4 kb and insert up to 3.0 kb of heterologous DNA [37]. A high-efficiency one-step integration strategy was developed in *E. coli*, with 70 to 100% efficiency at 7 different sites [38]. After optimizing the experimental conditions, the replacement efficiency of the *lacZ* gene using λ-Red protein and linear dsDNA was as high as 99%, and the efficiency of integrating 7.0 kb of foreign DNA was 61% [39]. Similarly, two large-fragment deletions were successfully introduced in *B. subtilis* using a single-plasmid system [40]. In *Clostridium acetobutylicum* ATCC824, the integration of large fragments was achieved using the CRISPR/Cas9 double plasmid system, with gene deletions and insertions of up to 3.6 kb [41].

In further work, researchers optimized the system to simultaneously edit multiple genes. A CRISPR/Cas system for *Streptomyces* was designed as a rapid multiplex genome editing tool, enabling targeted chromosomal deletions of 20 to 30 kb, with efficiencies from 70 to 100% [42]. Additionally, CRISPR/Cas9 knock-in technology was developed to activate biosynthetic gene clusters (BGCs) in *Streptomyces*. Researchers used this technology to successfully increase the production of specific metabolites in five *Streptomyces* species [43].

The lack of suitable genome editing tools has largely limited the development and utilization of some industrial strains, but the situation has changed dramatically with the emergence of the CRISPR/Cas9 system. In fact, diverse species including *Clostridium acetobutylicum* [44], *Synechococcus elongatus* UTEX 2973 [45], *Actinoplanes* sp. [46], *B. subtilis* [47], *Corynebacterium glutamicum* [48–50], *Clostridium beijerinckii* [51], *Streptomyces* sp. [52, 53], *Clostridium difficile* [54], *Clostridium saccharoperbutylacetonicum* N1–4 [55], and *Clostridium autoethanogenum* [56], have all been successfully engineered in recent years (Table 2).

**Table 2:** Applications of type II CRISPR/Cas systems in bacteria, including genome editing, transcriptional regulation and base editors

Cas protein	Target species	Strategy and type of modifications	References
Sp Cas9	*Actinomycetes*	Genome editing, deletion and replacement	[58]
Sp Cas9	*Actinoplanes* sp.	Genome editing, deletion	[46]
Sp Cas9	*B. subtilis*	Genome editing, deletions (25.1 kb and 4.1 kb)	[40]
Sp Cas9	*B. subtilis*	Genome editing, gene disruption (33 to 53%)	[47]
Sp Cas9	*C. acetobutylicum*	Genome editing, deletions and insertions (3.6 kb)	[41]
Sp Cas9	*C. acetobutylicum*	Genome editing, deletion and replacement	[44]
Sp Cas9	*C. autoethanogenum*	Genome editing, deletions (over 50% when screening a small library of tetracycline-inducible promoters)	[56]
Sp Cas9	*C. beijerinckii*	Genome editing, deletion and integration in single steps	[51]
Sp Cas9	*C. difficile*	Genome editing, site-specific mutations (20–50%)	[54]
Sp Cas9	*C. glutamicum*	Genome editing, deletion, point mutations and insertion (up to 100%)	[50]
Sp Cas9	*C. glutamicum*	Genome editing, knockout and GABA overproduction	[48]
Sp Cas9	*C. glutamicum*	Genome editing, deletion (60%) and insertion (62.5%)	[49]
Sp Cas9	*C. saccharoperbutylacetonicum*	Genome editing, deletions (75%)	[55]
Sp Cas9	*E. coli*	Genome editing, knockouts, insertions or substitutions (100%, 5 days)	[28]
Sp Cas9	*E. coli*	Genome editing, point mutations, deletions, and insertions	[29]
Sp Cas9	*E. coli*	Genome editing, knock-in	[30]
Sp Cas9	*E. coli*	Genome editing, knockout (100%, 3 days)	[31]
Sp Cas9	*E. coli*	Genome editing, (3 genes between 96.5 and 99.7%)	[33]
Sp Cas9	*E. coli*	Genome editing, deletions, insertions, and replacements (100%)	[35]
Sp Cas9	*E. coli*	Genome editing, deletion (19.4 kb) and insertion (3 kb)	[37]
Sp Cas9	*E. coli*	Genome editing, deletion (large chromosomal DNA fragments)	[57]
Sp Cas9	*E. coli*	Genome editing, insertion (70 to 100%)	[38]
Sp Cas9	*E. coli*	Genome editing, replacement (99%) and insertion (2.4 kb 91%, 3.9 kb 92%, 5.4 kb 71%, and 7.0 kb 61%)	[39]
Sp Cas9	*S. aureus*	Genome editing, knockout, knock-in and single base mutations	[32]
Sp Cas9	*S. coelicolor*	Genome editing, deletion (939 bp)	[53]
Sp Cas9	*S. coelicolor*	Genome editing, single gene deletion, single large-size gene cluster deletion (60% to 100%), simultaneous deletions of actII-orf4 and redD, as well as the ACT and RED biosynthetic gene clusters with high efficiencies of 54 and 45%, respectively.	[34]
Sp Cas9	*S. elongatus*	Genome editing, deletion (100%)	[45]
Sp Cas9	*Streptomyces*	Multiple genome editing, deletions (from 20 bp to 30 kb, 70 to 100%)	[42]
Sp Cas9	*Streptomyces*	Multiple genome editing, knock-in (5 species)	[43]
Sp Cas9	*S. rimosus*	Genome editing, deletions (100%) and point mutations	[52]
Thermo Cas9	*B. smithii*	Genome editing, knockouts and silencing (55 °C)	[36]
Sp nCas9	*B. licheniformis*	Genome editing, deletions (1 gene 100%, 2 genes 11.6%, large-fragment 79%) and insertions (76.5%)	[61]
Sp nCas9	*C. perfringens*	Genome editing, deletion (23 bp)	[62]
Sp nCas9 (D10A)	*E. coli*	Genome editing, deletions (from 36 to 96 kb)	[17]
Sp nCas9 (D10A)	*L. casei*	Genome editing, deletions and insertions (25 to 62%)	[59]
Sp dCas9	*B. subtilis*	CRISPRi, investigation of gene function (289 known or proposed essential genes, ~ 94% successfully targeting of bona fide essential genes)	[69]
Sp dCas9	*C. glutamicum*	CRISPRi (single gene, two genes)	[63]
Sp dCas9	*C. acetobutylicum*	CRISPRi	[60]
Sp dCas9	*C. beijerinckii*	CRISPRi (97%)	[64]
Sp dCas9	*E. coli*	CRISPRi	[23]
Sp dCas9	*E. coli*	CRISPRi (1000-fold repression)	[24]
Sp dCas9	*E. coli*	CRISPRi (10-fold repression)	[21]
Sp dCas9	*E. coli*	CRISPRi	[22]
Sp dCas9	*E. coli*	CRISPRi, investigation of gene function	[68]
Sp dCas9	*E. coli*	CRISPRi, harboring a biosynthetic mevalonate (MVA) pathway and enhancing production of (-)-α-bisabolol (C15) and lycopene (C40)	[71]
Sp dCas9	*E. coli*	CRISPRi, pinosylvin biosynthesis by inactivating a malonyl-CoA depleting pathway and a 1.9-fold increase of the pinosylvin content	[73]
Sp dCas9	*E. coli*	CRISPRi, pinosylvin synthesis pathway and the final pinosylvin titer was improved to 281 mg/L, which was the highest pinosylvin titer	[119]
Sp dCas9	*E. coli*	CRISPRi, the methionine biosynthetic pathway and a final titer of 51 mg/L(21-fold improvement overall)	[74]
Sp dCas9	*E. coli*	CRISPRi, malate biosynthetic pathway and 2.3-fold increase in malate titer	[75]
Sp dCas9	*E. coli*	CRISPRi, multiplex repression of competing pathway and n‑butanol yield and productivity increased up to 5.4‑ and 3.2‑fold, respectively.	[76]
Sp dCas9	*E. coli*	CRISPRi, downregulate fatty acid biosynthesis pathway to inactivate the malonyl-CoA consumption pathway	[77]
Sp dCas9	*E. coli*	CRISPRi, 1,4-BDO production and enhanced the 1,4-BDO titer for 100% to 1.8 g/L	[78]
Sp dCas9	*E. coli*	CRISPRi, the butanol synthetic pathway and 0.82 g/L butanol production	[79]
Sp dCas9	*E. coli*	CRISPRi, the biological synthesis of polyketides, flavonoids and biofuels and 7.4-fold higher production	[80]
Sp dCas9	*M. tuberculosis*	CRISPRi	[67]
Sp dCas9	*M. tuberculosis*	CRISPRi, single or multiple targets	[66]
Sp dCas9	*Pseudomonas* spp.	CRISPRi	[20]
Sp dCas9	*B. melitensis*	Base editor (C-T, 100%)	[26]
Sp dCas9	*C. glutamicum*	Base editor, (single-locus, 100%, double-locus, 87.2%, and triple-locus, 23.3%)	[85]
Sp dCas9	*E. coli*	Base editor (C-T, 99.93%)	[26]
Sp dCas9	*K. pneumoniae*	Base editor (position, PAM distal 4 to 8 bp, efficiency 100%)	[87]
Sp dCas9	*Staphylococcus*	Base editor (position, PAM distal 4 to 8 bp, efficiency 100%)	[88]

#### CRISPR/Cas9 and NHEJ-mediated genome editing

In spite of the low efficiency of HDR in some species, genome editing can be achieved by introducing a recombinant plasmid containing an exogenous NHEJ system. A corresponding gene-editing method was implemented in *E. coli*, which could delete large DNA fragments in one step without the need for a homologous DNA template [57].

Researchers used CRISPR/Cas9 technology to specifically induce DSBs in actinomycetes and repaired the resulting blunt ends using the error-prone NHEJ pathway, resulting in insertions or deletions at the target site [58].

### CRISPR/nCas9-mediated genome editing

Because of the lack of highly efficient genetic manipulation tools for *Lactobacillus casei*, single-gene knockouts were recently still being performed using the classical HDR-dependent double exchange method, which requires at least 24 days. To overcome this, a CRISPR/nCas9 (D10A) system was developed as a rapid and precise genome editing tool for *L. casei* [59]. Effective single-gene deletions and insertions were achieved in 9 days, contributing to the fast and accurate genome editing of *L. casei*.

In *E. coli*, the CRISPR/nCas9 system could be used to form non-lethal single-strand nicks, conduct precise editing of targeted genes, and successfully delete genomic fragments with a size from 36 to 96 kb. Moreover, multiple targeting was used to delete 133 kb [17]. Researchers used the CRISPR/nCas9 and HDR systems to achieve genome editing in *Clostridium acetobutylicum* ATCC 824 and *Clostridium beijerinckii* NCIMB 8052, with the highest efficiency reaching 100% [60].

In *Bacillus licheniformis*, the CRISPR/nCas9 system was used to successfully delete the *yvmC* gene with a remarkable editing efficiency of practically 100%. However, the efficiency of simultaneously editing of two genes was only 11.6%. Nevertheless, the efficiency of large-fragment deletion was 79.0%, and the insertion efficiency of the heterologous *aprN* gene for the expression of nattokinase reached 76.5% [61]. In *Clostridium perfringens*, application of the CRISPR/Cas9 system resulted in cell death. Nevertheless, the system was used to perform precise editing on the expected locus with an editing efficiency of over 95% [62]. These studies offer valuable resources for genome editing in bacteria (Table 2).

### CRISPR/dCas9-mediated CRISPRi

CRISPRi technology, which is based on dCas9, can effectively inhibit the expression of target genes in a number of bacteria, such as *C. glutamicum* [63], *C. acetobutylicum* [60], *C. perfringens* [60], *C. beijerinckii* [64], *Pseudomonas* sp. [20], *B. subtilis* [65], *Mycobacterium tuberculosis* [66, 67], and *E. coli* [21–24].

CRISPRi technology has important applications in the investigation of gene function, since it can be used to reversibly knock down the expression of target genes. Therefore, this technology provides an effective method to identify the function of essential genes and is increasingly being used to characterize genes with hitherto unknown functions in bacteria such as *E. coli* [68], *B. subtilis* [69], and *M. tuberculosis* [67].

Some bacteria are important industrial microorganisms for the production of various chemicals, but a lack of effective genetic tools may cause bottlenecks in metabolic engineering. Therefore, the application of CRISPRi technology in the transformation of metabolic pathways can effectively increase production. CRISPRi technology was used to inhibit genes in *C. glutamicum* and determine the effect of target-gene inhibition on amino acid titers [70]. SgRNAs were used to direct dCas9 to *pgi* and *pck*, which reduced their expression by 98 and 97%, respectively.

Numerous studies have shown that the CRISPRi system can be used to fine-tune the biosynthetic pathways in *E. coli* to increase the yield of target products, such as terpenoids [71], controllable P(3HB-co-4HB) [72], pinosylvin [73, 119], methionine [74], malic acid [75], n-butanol [76], resveratrol [77], 1,4-butanediol (1,4-BDO) [78], butanol [79], and flavonoids [80] (Table 2).

### CRISPR/dCas9-mediated base editor

Many bacteria can be genetically modified using CRISPR/Cas9 systems, but DSBs introduced by the Cas9 nuclease are severely toxic to some species. The CRISPR/dCas9-mediated base editor is independent of the host cell’s own NHEJ or HDR pathways. Instead utilizing the targeting effect of CRISPR/dCas9 to guide deaminase and induce mutations at specific sites [81, 82]. There are currently two main base editors: a cytosine base editor (CBE) based on cytosine deaminase, which can realize base changes from cytosine to thymine (C to T) [83], and an adenine base editor (ABE) based on adenine deaminase, which can realize base changes from adenine to guanine (A to G) at the target site [84].

The study of base editors in prokaryotes is relatively new and there are few reports. In 2018, Kondo et al. [18] used the CBE editing tool to implement the C to T base mutation in *E. coli* for the first time In the same year, researchers proved that the CBE editing system can induce C to T mutations in bacteria such as *Corynebacterium glutamicum* [85] *Pseudomonas aeruginosa*, *Pseudomonas aeruginosa*, *Pseudomonas putida* and *Pseudomonas fluorescens* [86]. By mutating the codons encoding amino acids to stop codons, the researchers successfully inactivated the corresponding genes in *E. coli* [18], *C. glutamicum* [85], *Klebsiella pneumonia*e [87], and *Staphylococcus au*reus [88], which proved that the CBE tool can achieve gene inactivation. At present, the ABE base editor is only used to realize A to G base changes in *E. coli* [84, 89]. The biggest advantage of the base editor compared with CRISPR/Cas9-guided gene editing is that it does not cause bacterial death, but a major disadvantage is that it can only induce base replacement, leading to mutation or gene inactivation, and cannot be used for gene insertions or deletions. This may also limit its development in prokaryotes.4 Applications of type V CRISPR/Cas systems in bacteria.

Type V CRISPR/Cas systems include ten subtypes, termed V-A to V-I and V-U. Among them, Cas12a (also known as Cpf1) was the earliest to be characterized and is the only one used as an editing tool in bacteria [90]. Cas12a was developed as a novel genome-editing tool, which expands the types of nucleases available for genetic editing of bacteria. Compared to Cas9, it has a number of advantages. First, Cas12a recognizes a T-rich PAM, extending the range of applications for genome editing tools. Secondly, the off-target rate of Cas12a is lower [91]. Thirdly, the guide-RNA of Cas12a is a single crRNA, which simplifies the process of multiplex editing by serially expressing multiple crRNAs.

### CRISPR/Cas12a-mediated genome editing

#### CRISPR/Cas12a and HDR-mediated genome editing

AsCas12a from *Acidaminococcus* sp. was used for genome editing in *C. beijerinckii*, an important species for the production of biosolvents via the acetone-butanol-ethanol pathway [92]. This provided a key reference for using the CRISPR/Cas12a system for genomic engineering. In *S. coelicolor*, the *actlorf1* and *redX* genes were successfully knocked out using HDR in conjunction with CRISPR/Cas12a technology, and the single-gene editing efficiency ranged from 90 to 95% [93].

A CRISPR/Cas12a-based genome editing tool achieved multiple genome editing with high efficiency and was the first system that was applied for multiple genome editing in *C. difficile* [94]. Using the CRISPR/Cas12a system to promote recombination via dsDNA cleavage together with λ-Red recombinase, up to 3 heterologous genes were simultaneously inserted into multiple sites of the *E. coli* genome [95].

Although the *S. pyogenes* (Sp) CRISPR/Cas9 system has been used in various bacteria, expressing the system in some species results in bacterial cell death [96]. Hence, it is necessary to use CRISPR/Cas12a-mediated genome editing technology for some bacteria. In *C. glutamicum*, researchers successfully induced a 50 bp deletion in the *crtYf* gene through CRISPR/Cas12a-assisted recombination engineering, with an editing efficiency of approximately 15%. Moreover, the editing efficiency for a 17 bp deletion increased to 40%, and that of 2 nucleotide substitutions in *argR* was 100%. However, this method failed to produce a 500 bp deletion (Table 3).

**Table 3:** Applications of type V CRISPR/Cas systems in bacteria, including genome editing and transcriptional regulation

Cas protein	Species	Strategy and type of modifications	Reference
FnCas12a	*C. glutamicum*	Genome editing, 2 nucleotide substitutions 100%	[96]
FnCas12a	*E. coli*	Genome editing, 3 heterologous genes were simultaneously inserted (20%)	[95]
FnCas12a	*S. coelicolor*	Genome editing, knocked out (100%)	[93]
AsCas12a	*C. difficile*	Multiple genome editing	[94]
FndCas12a (D917A)	*Y. lipolytica*	CRISPRi (85%)	[100]
AsdCas12a (E993A)	*E. coli*	Multiplex gene regulation	[98]

#### CRISPR/Cas12a and NHEJ-mediated genome editing

A single plasmid encoding CRISPR/FnCpf1 from *Francisella tularensis* and NHEJ completed N iterations of genome editing in 7 N + 2 days, and the efficiency was as high as 70%. Therefore, the system can greatly decrease the genome manipulation time required for *Mycobacterium smegmatis* [97].

Researchers selected three NHEJ systems from *M. smegmatis*, *Streptomyces daghestanicus*, and *Pseudomonas putida*, respectively named Msm-LK, Sda-LK, and Ppu-LK. These three systems promoted the repair of FnCpf1-induced DSBs, which successfully achieved DNA deletions of the desired size [93].

### CRISPR/dCas12a-mediated CRISPRi

The CRISPR/dCas9 system can regulate transcription, but it requires a cumbersome experimental procedure for multiplex editing. To solve this problem, the researchers mutated the glutamic acid at position 993 in the Cas12a protein to alanine and obtained DNase-inactivated CRISPR/dCas12a, which was successfully used for multi-site transcriptional regulation [98]. Similarly, researchers generated the mutant Cas12a (D917A), which can also be used in CRISPRi technology to regulate gene transcription [99, 100] (Table 3).

## Applications of type I and III CRISPR/Cas systems in bacteria

Although CRISPR/Cas9 and CRISPR/Cas12a systems have been extensively developed in most bacteria, they do not work in some species. As a result, some researchers have developed the endogenous CRISPR/Cas systems to edit bacterial genomes (Table 4).

**Table 4:** Applications of type I and III CRISPR/Cas systems in bacteria, including genome editing and transcriptional regulation

Cas protein	Species	Strategy and type of modifications	Reference
Cas3	*C. tyrobutyricum*	Genome editing, single- and multi-gene deletions (100%)	[103]
Cas3	*E. coli*	Genome editing	[106]
Cas3	*H. hispanica*	Genome editing, deletion and single nucleotide substitution	[102]
Cas3	*H. volcanii*	CRISPRi, the promoter region (down to 8%), the coding strand (down to 88%), the template strand (down to 8%)	[101]
dCas3	*E. coli*	CRISPRi (82%)	[105]
Cas10	*S. aureus*	Genome editing, deletions and insertions	[10]

### Genome editing using the endogenous type I CRISPR/Cas systems

Type I CRISPR/Cas systems include seven subtypes, I-A to I-F and I-U. Cas8a, Cas8b, and Cas8c are signature proteins of the I-A, I-B, and I-C systems, respectively. Similarly, Cas10d is the signature protein of the I-D subtype, while Cse1 and Cse2 are signature proteins of the I-E subtype. I-F includes four signature proteins, Csy1, Csy2, Csy3, and Csy6. In addition, the letter U in the designation of the I-U subtype represents a signature protein of unknown function. However, recent reports indicate that only I-B and I-E CRISPR/Cas systems have been developed as genome editing tools in bacteria.

The typeI-B system uses multiple Cas proteins in conjunction with mature crRNA to form a CRISPR-related antiviral defense complex (Cascade) to target and guide Cas3 protein to cleave foreign DNA fragments. Researchers demonstrated that the endogenous I-B CRISPR/Cas system can be used to inhibit gene expression in *Haloferax volcanii* [101]. Accurate genome editing in the polyploid halophilic archaeon *Haloarcula hispanica* was performed using the endogenous I-B CRISPR/Cas system [102]. The results showed that this system can easily simultaneously edit two target sites. Single- and multi-gene deletions were successfully performed using the endogenous I-B CRISPR/Cas system of *Clostridium tyrobutyricum* with an editing efficiency of 100% [103]. All these studies have showed the broad applicability of endogenous CRISPR/Cas systems in their native bacterial hosts. The heterologously expressed I-B system/Cas derived from *Methanococcus maripaludis* can inhibit the invasion of phage λ in *E. coli*, demonstrating the potential of the heterologously expressed I-B CRISPR/Cas system for gene manipulation [104].

A CRISPRi system was constructed in *E. coli* by deleting Cas3 protein with a cleavage effect and expressing the CRISPR-associated complex with a targeting effect. Using green fluorescent protein as a reporter, it was demonstrated that the endogenous I-E CRISPR/Cas system can downregulate target gene expression by 82% in *E. coli* [105]. Using the modified endogenous I-E CRISPR/Cas system, six different genes can be targeted simultaneously, which was used to screen mutants that increase the flux of malonyl-CoA for improved 3-hydroxypropionic acid (3HP) production in *E. coli* [106]. This method provided a rapid and simple strategy for regulating metabolic pathways and modifying industrial strains. The endogenous I-E CRISPR/Cas system was developed for insertions, deletions, and single-base substitutions in *Lactobacillus crispatus*, which expanded the CRISPR toolbox [10]. These studies demonstrate that the endogenous I-E CRISPR/Cas system is a simple and powerful tool of regulating metabolic fluxes.

### Genome editing using endogenous type III CRISPR/Cas systems

The type III CRISPR/Cas systems include four subtypes, III-A to III-D. Among them, the III-A subtype contains Csm series proteins, Cas1, Cas2, and Cas6 proteins. Csm is primarily a crRNA-guided RNA nuclease, but it also has DNase and cyclic oligoadenylate (cOA) synthetase activities [107]. The III-B subtype contains Cmr series proteins but lacks Cas1, Cas2, and Cas6 proteins. Cmr recognizes and degrades DNA or RNA based on the complementarity of crRNA sequences [108], so it depends on other CRISPR systems in the organism when interfering with RNA. The III-C subtype contains a cyclase domain-inactivated Cas10 protein. The III-D subtype contains an unknown functional gene, and the Cas10 protein of this subtype lacks the HD domain [109].

By constructing the III-A CRISPR/Cas system modules from three bacterial species and heterologously expressing them in *E. coli*, it was found that expression modules from *Streptococcus thermophilus*, *Lactococcus lactis* and *Staphylococcus epidermidis* specifically eliminated an invasive plasmid recognized by the crRNA, which provided a new direction for the study of the III-A CRISPR/Cas system in *E. coli* [11].

The *S. aureus* type III-A system can achieve large-fragment genomic deletions and insertions [110]. A truncation of 10–13 nucleotides in the spacer blocked the CRISPR attack, and truncations of more than 13 nucleotides completely eliminated targeting. These results suggest that the type III-A system regulates the stability of the bacterial genome and can be used as an efficient tool for gene knockout in bacteria.

## Prospects of CRISPR technology

Currently, the CRISPR/Cas tools used in bacteria still face challenges, such as the high off-target rate of Cas9, weak cleavage activity of Cas12a, and insufficient development of endogenous systems. Therefore, in view of the problems and challenges, we offer some perspectives in the following aspects.

To reduce the off-target rate, researchers modified the Cas9 protein by replacing positively charged residues with neutral amino acids, and obtained eSpCas9 (K810A/K1003A/R1060A or K848A/K1003A/R1060A) [111] as well as a new hyper-accurate Cas9 variant (N692A/M694A/Q695A/H698A, named HypaCas9) with no effect on targeted activity [112]. Furthermore, in order to improve cleavage activity, researchers focused on the CRISPR/Cas12a systems based on AsCas12a and LbCas12a, and inserted a HDV ribozyme at the 3′ end of the crRNA [113], which increased the editing efficiency by 1.1- to 5.2-fold. To further expand the editing toolkit, researchers optimized existing Cas proteins, such as AsCas12a (enAsCas12a) [114] and FnCas12a [115], which can identify a wide range of PAM sequences. Additionally, researchers characterized new so-called CasX proteins, such as Cas12b and Cas12e. Cas12b showed a lower frequency of off-target effects in eukaryotes, along with a broader PAM sequence specificity [91, 116]. Recently, CasX was identified as a new RNA-directed DNA endonuclease in *E. coli* that uses a specific structure to cleave targeted genes [117].

In bacteria, the main single-base editor type is CBE, which causes a termination of gene expression and inactivation of protein function by introducing a stop codon. This approach greatly simplifies functional gene identification and metabolic engineering studies. Phage-assisted continuous evolution of the base editor (BE–PACE) was established to increase the editing efficiency and target gene compatibility without GC target limitation [118]. However, it should be noted that the off-target rate of the current CBE systems used in bacteria is relatively high, and base editors can be optimized by carefully designing sgRNAs or protein engineering to reduce the off-target rate in the future.

## Conclusions

Bacteria are often used as cell factories for the production of valuable metabolites such as amino acids, antibiotics, and vitamins. However, these approaches require extensive genetic modification of bacteria, which relies on the availability of robust genetic engineering tools. The emergence of the CRISPR/Cas systems provided a number of new tools for genetic modification of bacteria. The Cas9 and Cas12a proteins have been developed into powerful tools for exploring the genetic mechanisms of bacteria, the optimization of metabolic pathways of industrial microorganisms, and other genetic modifications.

Different Cas proteins and DNA repair systems can be selected according to experimental needs to achieve efficient knockout or insertion of target genes. In bacteria that cannot express Cas9 and Cas12a nucleases due to toxicity, endogenous CRISPR/Cas systems have been developed for genetic manipulation. Additionally, base editors can be used to implement point mutations, while CRISPRi and CRISPRa can be used to regulate transcription. However, the available systems still have problems, and need to be optimized further. In addition, CRISPRa and ABE have yet to be widely developed in non-model bacteria, and the development of these technologies will further enrich the toolkits for genome editing, providing more options for the functional study of bacterial genomes.
